# Paired walkers with better first impression synchronize better

**DOI:** 10.1371/journal.pone.0227880

**Published:** 2020-02-21

**Authors:** Miao Cheng, Masaharu Kato, Jeffrey Allen Saunders, Chia-huei Tseng

**Affiliations:** 1 NTT Communication Science Laboratories, NTT Corporation, Atsugi, Japan; 2 Department of Psychology, University of Hong Kong, Hong Kong SAR, China; 3 Center for Baby Science, Doshisha University, Kyoto, Japan; 4 Research Institute of Electrical Communication, Tohoku University, Sendai, Japan; University of Minnesota, UNITED STATES

## Abstract

This study measured automatic walking synchronization and how it associates with social impression. Previous studies discovered positive social consequence of motor synchrony with ecological paradigms (e.g. body movement synchrony between therapists and patients in clinical sessions, and the synchrony of side-by-side walkers). However, most studies of joint movement with high ecological validity face the same challenge, namely that conversations between participants might be the main or a partial contributor to the observed social benefits, as conversation is well documented to promote understanding and motor synchronization. We addressed this issue by using a novel paradigm to remove the conversation component and examined how synchrony per se interacted with social impression. Participants were paired to walk side by side in silence (i.e. without conversation) and their social impression toward each other was rated before/after the paired walk. Our results showed that walkers' first impression was positively associated with their step synchronization rate in the silent paired walk. Together with past findings, the bi-directional relation between body entrainment and social functions suggests that implicit nonverbal communication plays a significant role in providing a basis for interpersonal interaction.

## Introduction

Human life frequently involves coordinating our movements with those of others–for example, marching in a group or walking side by side. Our movements can synchronize with others either consciously or unconsciously. In implicit synchrony, actors do not share an explicit goal to move synchronously; while in explicit synchrony, actors intentionally coordinated their movements in a synchronous pattern. Explicit motor synchrony occurs in situations where individuals voluntarily coordinate action to be synchronized and actors are aware of the synchronicity, such as when marching or dancing, or during musical performance. Motor synchrony can also be unintentional and spontaneous. For example, people unconsciously synchronize steps with each other while walking side by side, or when audience involuntarily clap in synchrony. It is not yet known whether these two classes of motor synchronization share the same operational algorithms or neuronal mechanisms.

Existing research has suggested a bidirectional relationship between motor synchrony and social factors. On one hand, social factors shape motor synchrony. The way that partners perceive each other affects the amount of synchrony between them. People tend to synchronize more with partners who are attractive [1], likable [2], punctual [3], or who have a prosocial attitude [4]. Motor synchrony is also affected by whether two actors perceive each other as an ingroup member. Miles and colleagues [5] reported a higher level of movement synchrony between members of two different groups than between members of the same group. On the other hand, there is evidence that motor synchrony can have positive social consequences. Laboratory studies have found that motor synchrony can induce a good impression of a partner [6–9], feelings of affection [10], feelings of trust [7], prosociality [11, 12] and cooperation [13]. These studies reveal that social relations can affect motor synchrony, and that motor synchrony also has an important role in social relations.

Some studies that have used naturalistic contexts have discovered positive social consequences from body synchrony. For instance, Ramseyer and Tschacher [14] employed motion energy analysis to quantify body movement synchrony between psychotherapist and patient during therapy sessions. They discovered that nonverbal synchrony was associated with a high quality therapeutic relationship. Convergent psychotherapy research supports the view that body synchrony enhances both alliance and a positive patient-therapist relationship (see a review article, [15]). Body synchrony during natural conversation also promotes rapport in non-clinical settings. Cornejo et al. [16] conducted a similar motion energy analysis of recorded natural conversation within pairs, and they discovered that body synchrony was positively associated with interpersonal trust. Kato, Hirose and Kashino [17] examined the antecedents and consequences of walking step synchrony in a real-world scenario by pairing strangers meeting for the first time and having them walk outdoors while engaging in casual conversation. The impressions of the partners were rated before and after walking, and an improved impression after walking was correlated with step synchrony although the effect was reduced after considering individual differences (e.g. gender and autistic traits) [18]. The results suggest interpersonal synchrony can have social effects in real world settings, and are not limited to laboratory conditions with artificial tasks.

However, the previous naturalistic studies of interpersonal synchronization share a major confounding factor: conversation between partners. Conversation was included in all of the above studies because of the nature of the task (e.g. clinical therapy was by nature a talking therapy) or to keep the situation natural (e.g. strangers meeting for the first time). However, this creates a confounding factor because conversation is known to modulate both body synchrony and interpersonal impression. First, conversation is known to affect the social relationship of partners engaged in a joint action. The convergence of speech rates in conversation is related to partners’ competence and attractiveness judgments [19] and cooperation in the prisoner’s dilemma game [20]. Second, the content of a conversation can shape how well people synchronize with each other. Paxton and Dale [21, 22] found that arguments inhibited the interpersonal convergence of body movements more than less competitive conversations. Tschacher and colleagues [10] also observed effects of conversation content, but found that debate promoted greater levels of body synchrony than cooperative conversation. Interestingly, verbal interaction alone did not enhance synchrony between two participants while they were swinging handheld pendulums side by side compared with participants swinging pendulums but without conversation [23]. These studies suggest that conversation content modulates body synchrony, rather than the presence of verbal interaction.

In this study, we controlled the possible confounding effect of conversation by developing a paradigm to disassociate explicit verbal communication and implicit body coordination with a silent paired walk. We created a scenario where participants were made to believe that they were going to assist in a time perception study. They were paired and told to walk silently at first, and then to walk while chatting. They were asked to estimate the duration of the silent period and we collected the perceived durations. This design enabled us to remove the effect of verbal communication and examine the interplay between social impression and motor synchrony.

## Experiment 1: Main experiment

### Method

#### Participants

The sample size was estimated from the effect size in our previous experiment [18], which indicated a significant correlation (r = .190, n = 153, p = .019) between walking synchrony and an enhanced post-walk impression of the partner. We estimated a similar correlation of 0.2 in the current study. A sample size of 191 pairs was estimated by a power analysis using G*Power 3 [24], with an alpha of 0.05 and a power of 0.8. To maximize the data collection efficiency, we paired each participant with every other participant in a session. Because the number of motor sensors was limited, we recruited a maximum of 8 participants for each session (the actual attendance per session was 4–8 participants due to participant availability and no-shows).

In total, we recruited 67 participants (37 females; mean age 19.88 years, SD 2.56 years; mean AQ 20.45, SD 6.50; mean height 167.69 cm, SD 9.80 cm; mean weight 57.07 kg, SD 10.65 kg) and conducted five sessions per gender. No mixed gender session (female-male pair) was conducted. There were 194 pairs in total (116 female pairs). All participants provided written informed consent before the experiment began. They were told a cover story, namely that this experiment studied participants’ time perception under different conditions, to draw their attention away from the fact that their walking steps were being recorded, and the real purpose was disclosed to them at the end. All the procedures were approved by the Human Research Ethics Committee of the University of Hong Kong (Reference No. EA231012). All the methods were performed in accordance with the principles expressed in the Declaration of Helsinki as regards the treatment of participants.

#### Procedure

To conceal our interest in walking synchrony, we misled the participants into believing that the study examined how social interaction affected time perception. Upon arrival, the participants were instructed to complete a personal information form (including age, height, weight, and foot length) and an AQ questionnaire [25]. They also rated their first impressions of every other participant using the Interpersonal Judgment Scale (IJS) questionnaire [26]. When the first impression (IJS1) was filled, participants sat on chairs arranged in a way that they could see all other participants and the number labeled on each of them. They filled in the rating for each of them without a chance to talk or to know each other. At that time, they had no knowledge about the experiment design. Then we announced the order of pairing and explained that every participant was to walk with every other participant. They were required to walk silently with each partner for the first half of the walk (point A to B) and then to walk while engaging in free conversation for the second half (point B to A). After each walk, they reported the perceived duration.

After the instruction, the participants were led outdoors where they undertook a practice walk alone to familiarize themselves with the path and the procedure. The walking path was part of a quiet and barrier-free path made of hard concrete on the campus of the University of Hong Kong. The distance (between points A and B) was fixed at about 350 m, and it took participants approximately 3–4.5 minutes to complete each one-way trip. The participants estimated the duration of their practice walk after completing the first half (at point B), and the experimenter, who was waiting at the end of the practice path, gave them the correct duration as feedback, so that the participants could have a sense of the duration of the one-way walk. The practice walk ended after the participants returned to point A. No feedback was given in subsequent experimental sessions.

**Fig 1:**
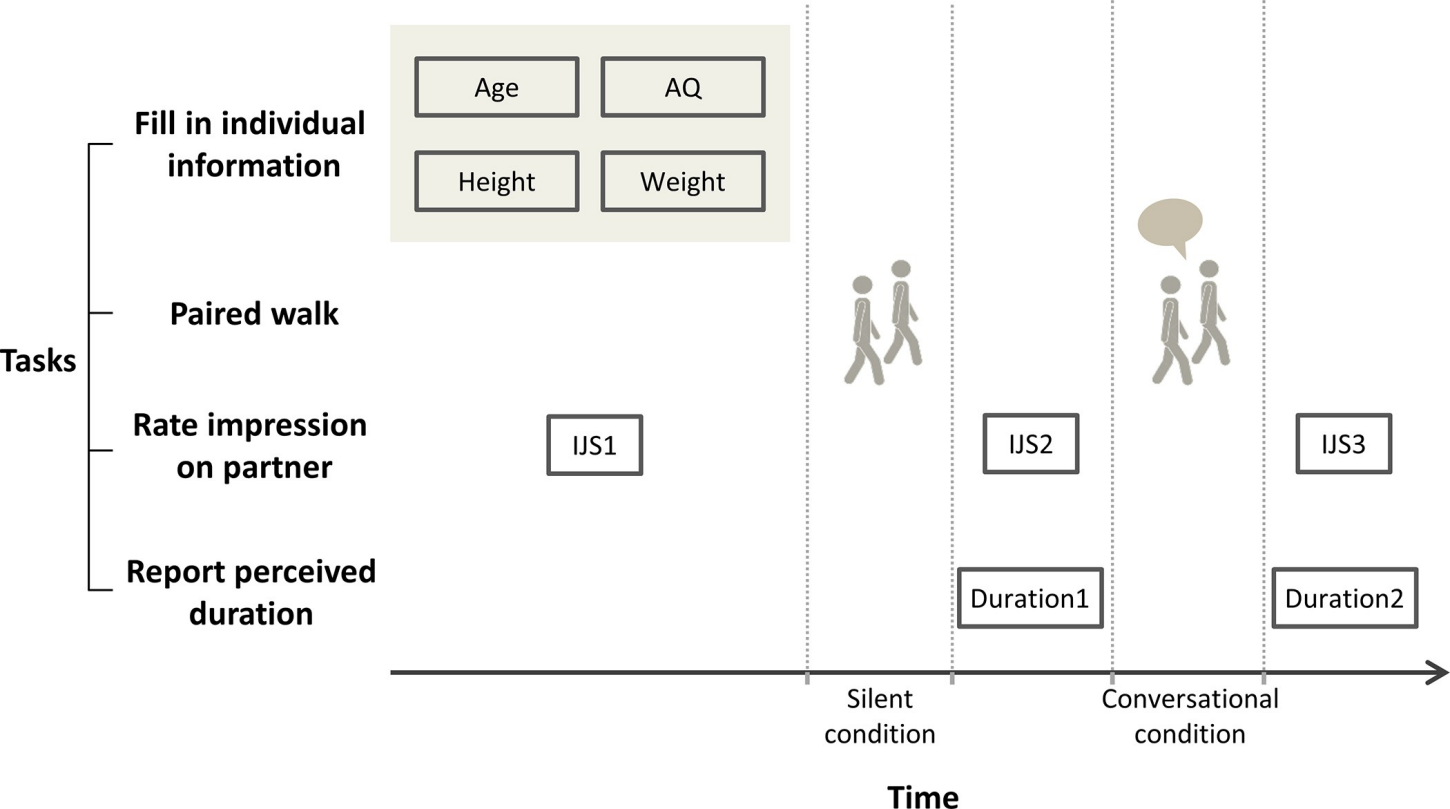
Tasks during the entire experiment.

After the practice walks, the participants were paired and asked to walk from point A to point B, side by side without any verbal communication (silent condition). From point B back to point A, they were allowed to chat while walking (conversational condition). After the silent condition, the participants estimated the time duration of the walk (duration 1) and again rated their impressions of their partners using the Interpersonal Judgement Scale (IJS2) at Point B; after the conversational condition, the participants reported the duration of the paired walk while conversing (duration 2) and again rated their impression (IJS3) once they had returned to point A. No feedback about duration was given to the participants in the paired walks. The procedure is shown in Fig 1. All participants always performed silent condition before conversational condition to avoid carryover effect from conversation, which was already reported in multiple studies previously including ours [17, 18]

During the walks, the participants wore voice recorders. Their walking movements were recorded by acceleration sensors (ATR-promotions, TSND121) attached above their right ankles and disguised as a GPS device. The sampling rate was 200 Hz. Recorded data were downloaded offline after the experiment. The sensors for each pair were time-locked by shaking them intentionally before the walk for coders to use later as a time marker for their synchronization calculation. To prevent any interference with their walking, the participants were asked to focus on the current task, to avoid bodily contact with each other, and to ignore distractions from the environment (e.g., mobile phones, friends passing by, or people asking for directions). The session continued until each participant had walked with every other participant. Each session was attended by 4–8 people (i.e. 6–28 pairs) and lasted 1–2.5 hours.

#### Data processing

We used the phase synchronization time ratio [PSTR, 17, 18], the ratio of the duration for which two walkers were phase-synchronized out of the total walking period, as an index for walking synchrony. All subsequent mathematical analyses were performed using Matlab (MathWorks, USA). Below is a brief summary of how the PSTR is constructed.

**Fig 2:**
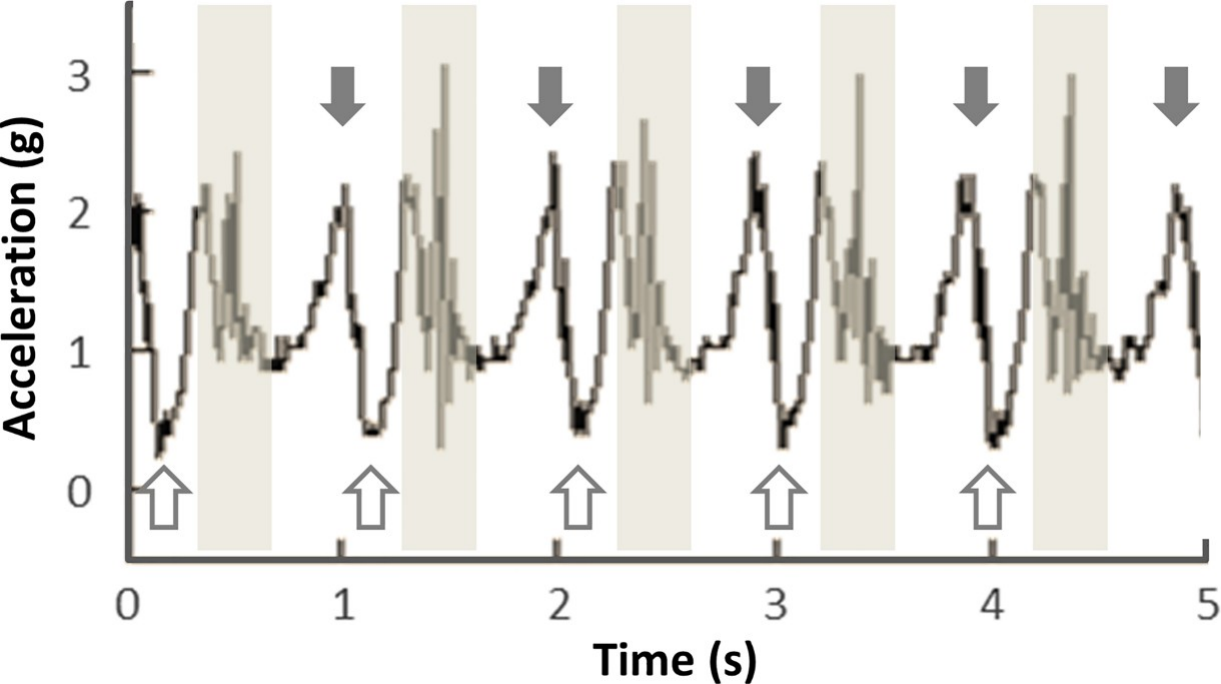
Example acceleration data extracted from a motion sensor. Peaks (filled arrows) represent the acceleration when the right foot was lifted, and the empty arrows represent the time during which the right foot was resting on the ground. The shaded areas show vibration noises that occurred when the right foot rested on the ground and the left foot was lifted.

Acceleration data were extracted from motion sensors strapped to the right ankles of the walkers. Fig 2 shows a 5-second recording obtained from a participant. In this example, the periodic curves indicate when the right foot was raised (filled arrows) and set down (open arrows). The vibration that occurred when the right foot was on the ground and the left foot was raised is also recorded in the graph (shaded zones). The distance between two peaks (produced by the same foot) was calculated to indicate the step duration, and we applied autocorrelation (to each individual’s acceleration data) to compute each participant’s step duration (or pitch, the inverse of frequency) for each 3.83 second time window with a window step of 20 ms. To obtain the lag between two paired walkers’ steps, we applied a cross-correlation analysis to two walkers’ acceleration data with a time window of 3.83 s and a window step of 20 ms. We derived each pair’s relative phase from their step duration, and a lag with a relative phase of 0 degrees indicated that paired walkers walked in-phase (i.e. heel strikes were synchronized between the ipsilateral legs of the walkers, e.g. right and right) and a relative phase of 180 degrees indicated anti-phase (i.e. heel strikes were synchronized between the contralateral legs of the walkers, e.g. right and left).

In addition, an order parameter, R was calculated based on the relative phase to account for the stability of synchronization [17]. R varied from 0 to 1, and an R value above 0.75 was defined as phase synchronization. We obtained the ratios of the total duration when R was above 0.75 (paired participants synchronized) relative to the total walking duration and called it the phase synchronization time ratio (PSTR) and converted it to percentages for easy reading in the subsequent analysis reports. With the assumption of a uniform distribution, the chance of R being above 0.75 is 25%.

### Results

A total of 37 female and 30 male participants formed 116 female pairs and 78 male pairs. 8 pairs were excluded due to hardware problems with the motion sensors. A total of 110 female and 76 male pairs were included in the data analysis.

#### Paired walkers synchronized even without conversation

To test whether walking synchrony occurred without its facilitation by conversation, we compared the PSTR between the silent and conversational conditions. We did not observe any gender difference as regards the PSTR (silent condition: female/male PSTR = 40.248, 37.449, t(184) = 1.694, p = .092; conversational condition: female/male PSTR = 40.033, 39.090, t(184) = 0.520, p = .604), so we combined data for male and female walkers for analysis.

**Fig 3:**
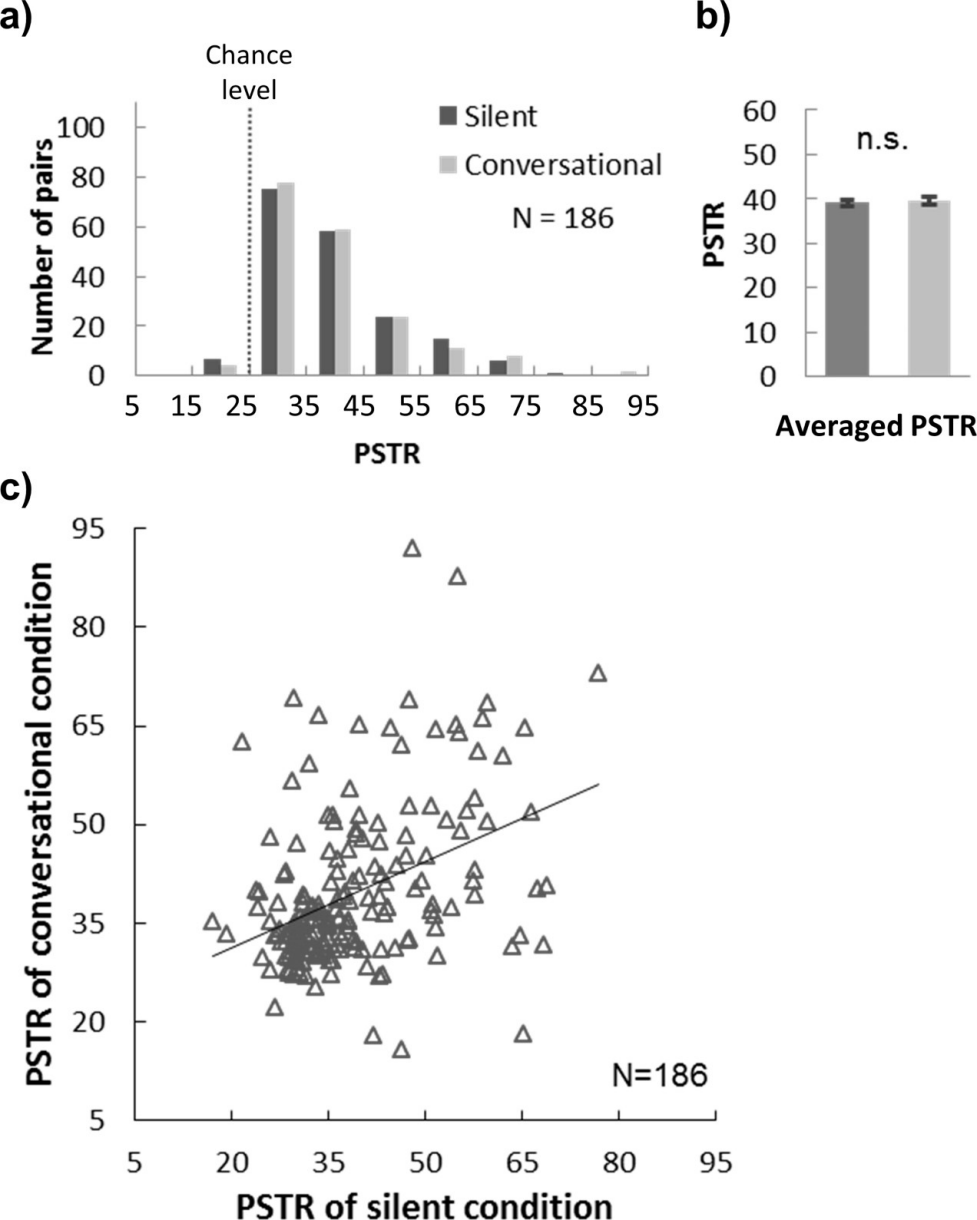
Walking synchrony under silent and conversational conditions. a) PSTR distribution under silent and conversational conditions. The chance level of PSTR (25) is shown by the dashed line. b) Means and 95% confidence intervals for PSTRs under the two conditions. c) Scatter plot between the PSTRs for silent and conversational conditions.

The PSTR distribution is summarized in Fig 3A. The average PSTRs for the silent (39.105) and conversational (39.648) conditions were both significantly above chance level (25) (silence: t(185) = 27.475, p < .001; conversation: t(185) = 25.790, p < .001) (Fig 3A). Walkers’ PSTR in the silent condition (i.e. without conversation) did not differ from that in the conversational condition (t(185) = 0.580, p = .563) (Fig 3B), contradicting the hypothesis that conversation is the key factor promoting step synchronization between paired walkers. Interestingly, each pair’s PSTRs under the two conditions were positively correlated (Fig 3C, r = .289, p = .011), indicating that walking synchrony between a pair remained stable in different contexts.

#### Walking without conversation improved impression of partner

**Fig 4:**
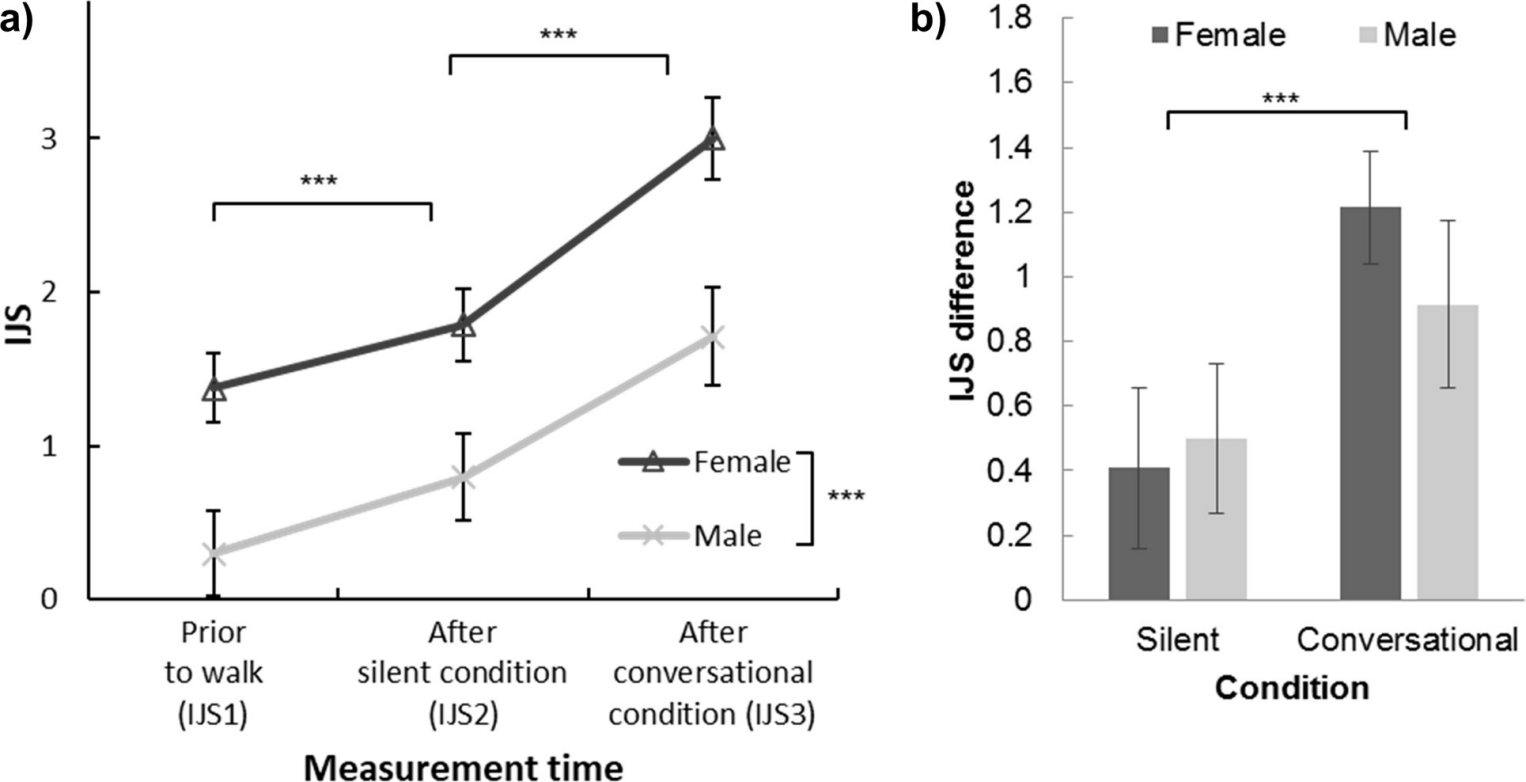
Results of impression rating for each gender. a) Means and 95% confidence intervals for female and male walkers’ total IJS scores measured before the silent condition (IJS1), after the silent condition (IJS2) and after the conversational condition (IJS3). b) IJS difference of each gender under silent (IJS2-IJS1) and conversational conditions (IJS3-IJS2) (***, p < .001).

To investigate whether or not a paired walk without conversation produced an impression improvement, we examined how the IJS scores changed under silent and conversational conditions (Fig 4). Because previous studies have shown a gender difference with regard to impression rating [18], we applied a two-way mixed design ANOVA to IJS scores with gender as a between-subject factor and measurement time (before the silent condition (IJS1), after the silent condition (IJS2) and after the conversational condition (IJS3)) as a within-subject factor. In general, females (2.055) gave significantly higher impression ratings than males (0.934), F(1, 184) = 46.895, p < .001, η^2^ = .203. A significant main effect of measurement time (F(2, 368) = 146.991, p < .001, η^2^ = .444) showed that the IJS rating varied during the experiments. The impression rating had improved significantly after each walk: a post-doc analysis with Bonferroni adjustments revealed that IJS2 (1.291) was significantly higher than IJS1 (0.837, p < .001); while IJS3 (2.355) was significantly higher than IJS2 (p < .001). We did not observe any significant interaction effect (p = .242). The above results indicated that even under the silent walk condition, the participants’ impression of their paired partner improved. Is the increment of silent condition comparable to the increment of conversational condition?

We obtained the IJS increment under the silent condition (by subtracting the mean of two walkers’ IJS1 scores from their mean IJS2 scores) and under the conversational condition (by subtracting IJS2 scores from IJS3 scores) (Fig 4B) and conducted a two-way ANOVA test with factors of gender as a between-subject factor and condition (silence and conversation) as a within-subject factor. A significant main effect of condition suggested that the IJS improvement under the conversational condition (1.064) was significantly greater than that under the silent condition (0.455, F(1, 184) = 20.786, p < .001, η^2^ = .102). The main effect of gender (p = .313) and the interaction (p = .146) were not significant.

Overall, females rated their partners more likable compared with males. For both genders, the impression of a partner became more favorable after paired walking without conversation (silent condition), and further increased after conversation had taken place. The impression improvement was greater for conversation than for silence.

#### Time perception and social impression

**Fig 5:**
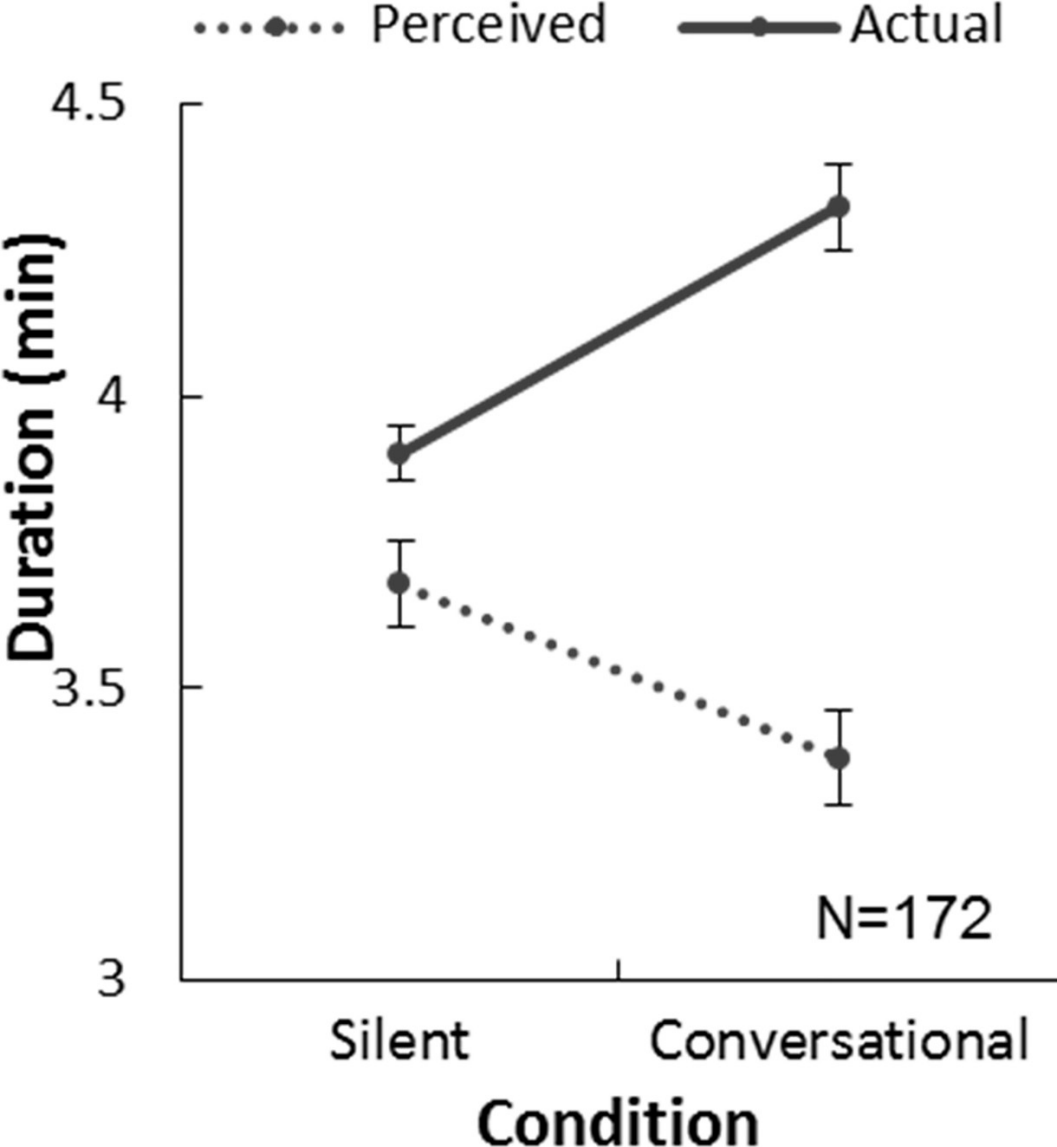
Perceived and actual duration of paired walk when silent and when conversing.

We analyzed the perceived and actual durations of paired walking under silent and conversational conditions. Due to data loss, only 172 pairs (103 female and 69 male pairs) were included in the analysis and the results are summarized in Fig 5. We averaged the two walkers’ perceived durations for each pair for analysis.

The participants underestimated the paired walking duration significantly more under the conversational condition than under the silent condition. We applied a two-way repeated measure ANOVA with factors of duration (perceived and actual durations) and condition (silence/conversation) for the paired walk (Fig 5). In general, the perceived duration (4.113 min) was significantly longer than the actual duration (3.529 min), F(1, 171) = 256.073, p < .001, η^2^ = .600. There was a significant interaction, F(1, 171) = 287.381, p < .001, η^2^ = .627. A post-hoc test showed that the time underestimation in the conversational condition was significantly greater than in the silent condition. The participants walked for 26 seconds longer when a conversation took place (4’20” vs. 3’54”, t(172) = 14.901, p < .001), but their perceived duration was 10 seconds longer than that under the silent condition (3’28” vs. 3’41”, t(172) = 9.183, p < .001).

**Fig 6:**
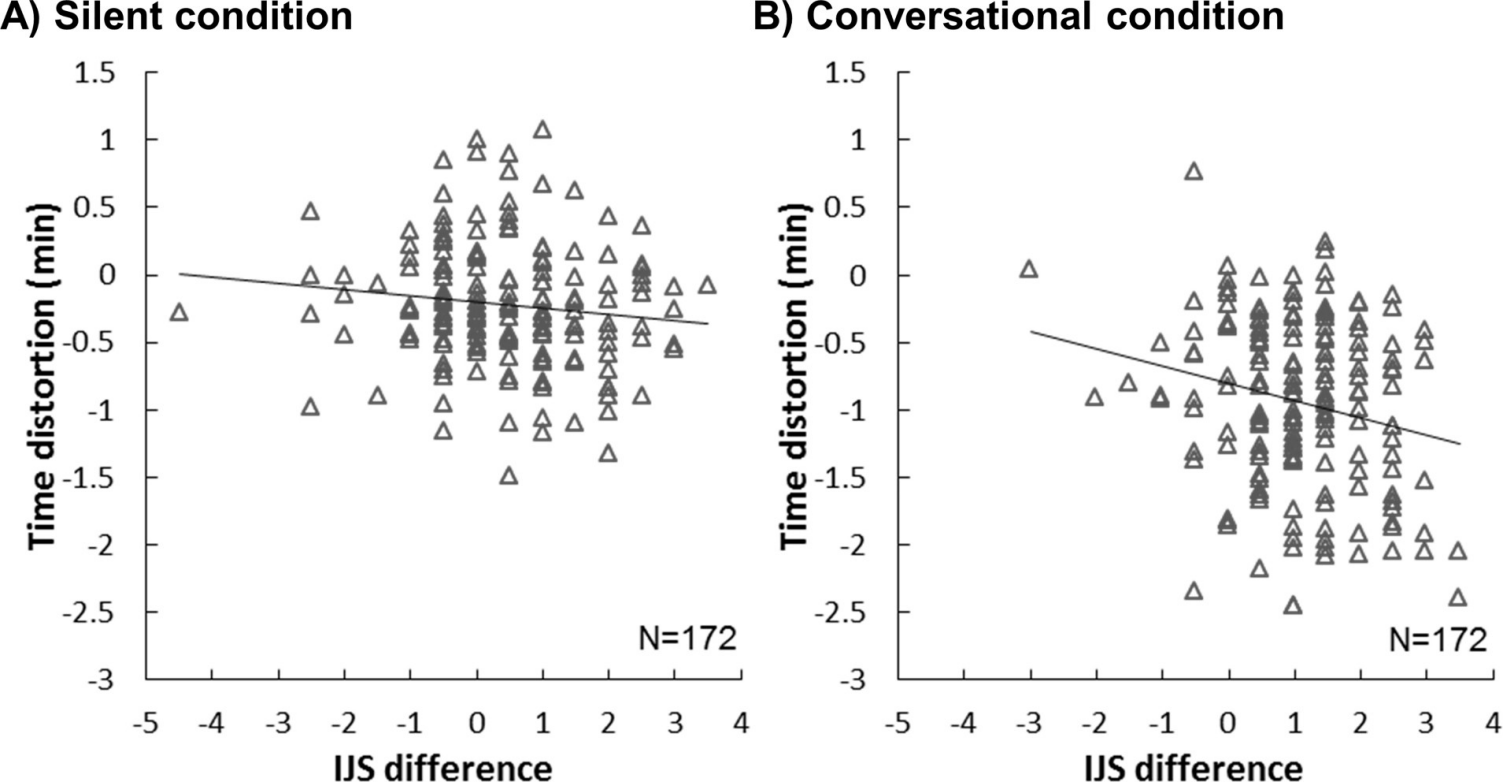
Time distortion and IJS difference when silent and when conversing.

It is known that time seems to pass more quickly when we are having fun [27] and we tested whether the participants’ time perception reflected their enjoyment of social interaction (e.g. did time pass more quickly when walking with a more likable partner?). We found that under the conversational condition only, the time distortion was greater when the participants showed a greater improvement in impression (Fig 6). For each pair, we obtained the time distortion of their paired walk by subtracting the perceived duration from the actual duration. A negative number indicates that the participants perceive time to be passing faster than its actual duration, and a positive number indicates time passing slower than its actual duration. We correlated the time distortion with the IJS difference for the silence and conversational conditions separately (Fig 6) and observed a significantly negative correlation for the conversational condition (r = -.209, p = .005) but not for the silent condition (r = -.122, p = .177).

The above results showed that 1) people walked slower and 2) they felt time passed more quickly when walking and engaging in conversation with partners than when walking in silence. 3) The underestimation of duration increased with a more likable partner.

#### Good first impression facilitated walking synchrony

**Fig 7:**
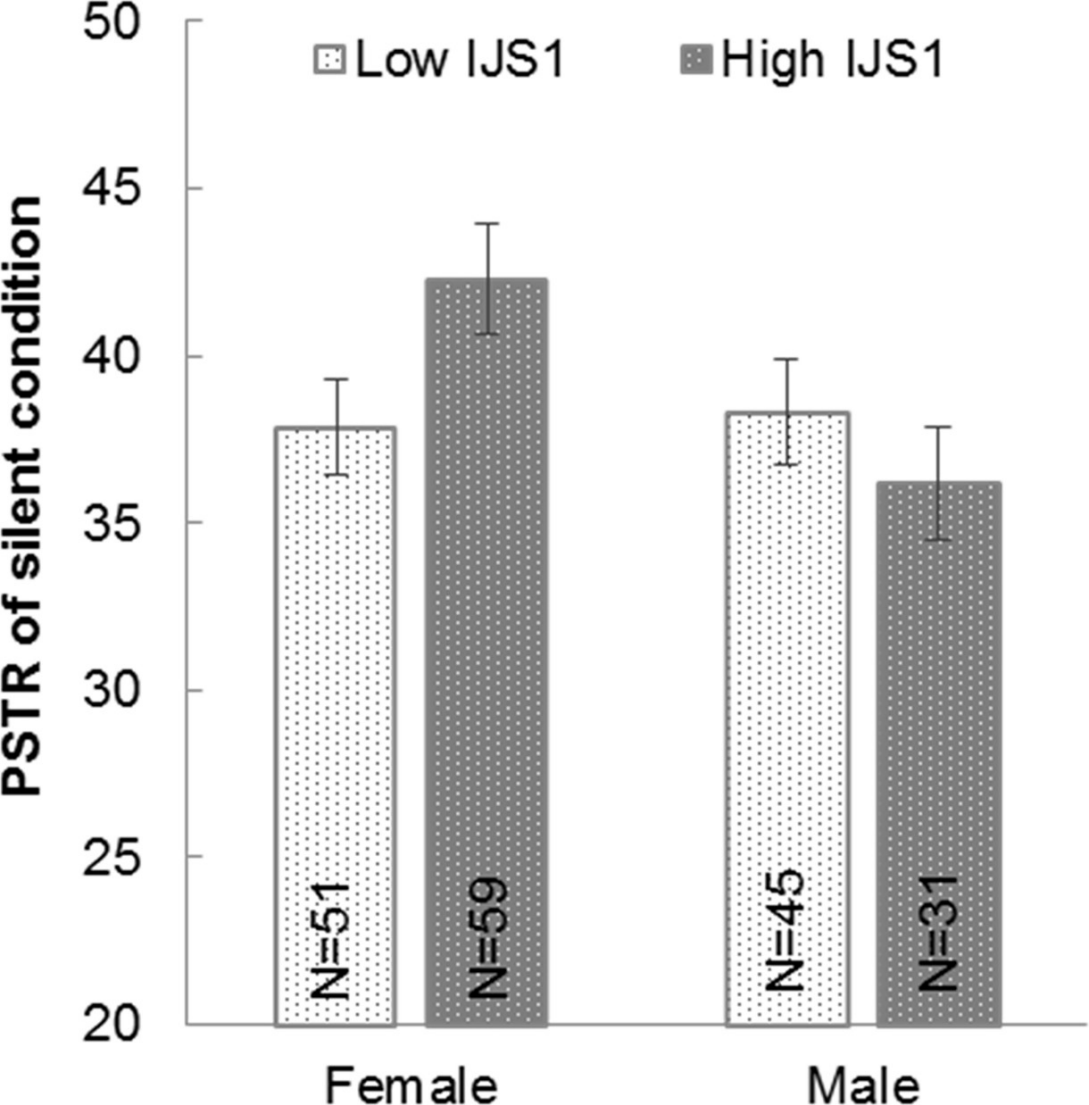
PSTRs of low and high IJS1 groups of each gender when walking in silence.

To investigate whether walking synchrony is associated with the social relationship between silent walkers, we first tested whether the first impression affected synchrony when walking in silence. We divided pairs into low and high first impression groups based on the IJS1 of each gender, and compared the two groups’ PSTR for the silent condition (Fig 7). Two-way ANCOVA with factors of gender and IJS1 group was applied to the silence PSTR, while controlling the mean age, mean AQ, height difference and weight difference of each pair, which had the potential to modulate walking synchrony [18, 28–30]. The results showed a marginally significant interaction effect between gender and IJS1 group, F(1, 178) = 3.592, p = .060, η^2^ = .020. A planned t-test analysis comparing the PSTRs of low and high IJS1 groups within each gender showed that with female walkers, the high first impression group (42.300) tended to synchronize more than the low group (37.874, t(108) = 2.012, p = .047); but for male walkers, the PSTRs of the low IJS1 (38.310) and high IJS1 (36.200) groups did not differ (p = .378).

**Fig 8:**
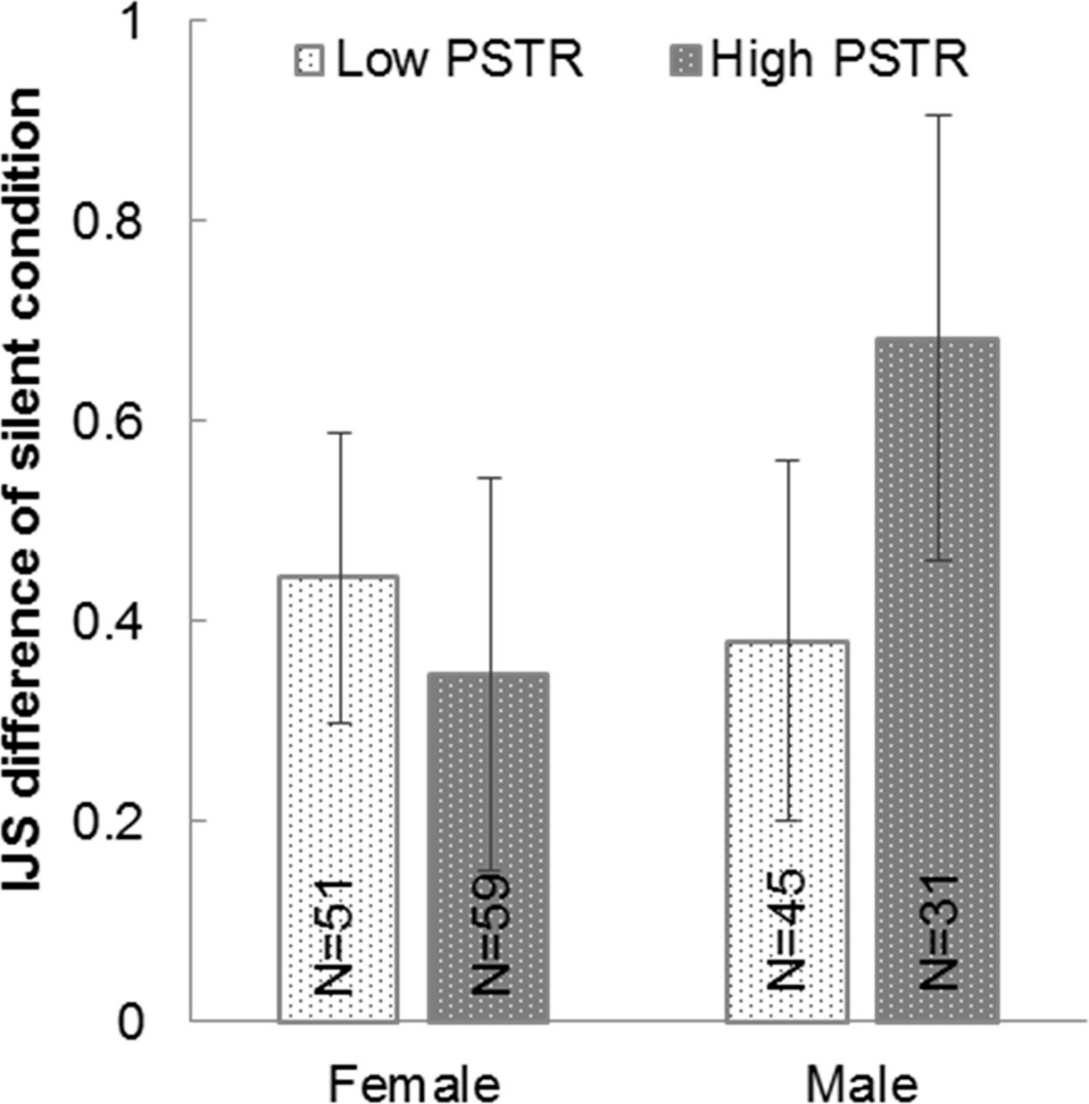
IJS difference between low and high PSTR groups obtained for each gender under the silent condition.

Did a silent pair’s walking synchrony affect the impression increment as with conversation [17, 18]? For each gender, we first classified participants into low synced and high synced groups based on the average PSTR obtained during silent walking (37.449 for males, 40.248 for females). We tested the IJS increment after silent paired walking (IJS2-IJS1) with a two-way ANOVA with factors of gender and PSTR groups (Fig 8). We did not observe any significant main effect (gender, p = .468; PSTR groups, p = .586) or interaction effect (p = .289), implying that walking synchrony was not significant in terms of impression change between two silent walkers, which is [inconsistent with previous studies when conversation takes place, 17, 18].

**Fig 9:**
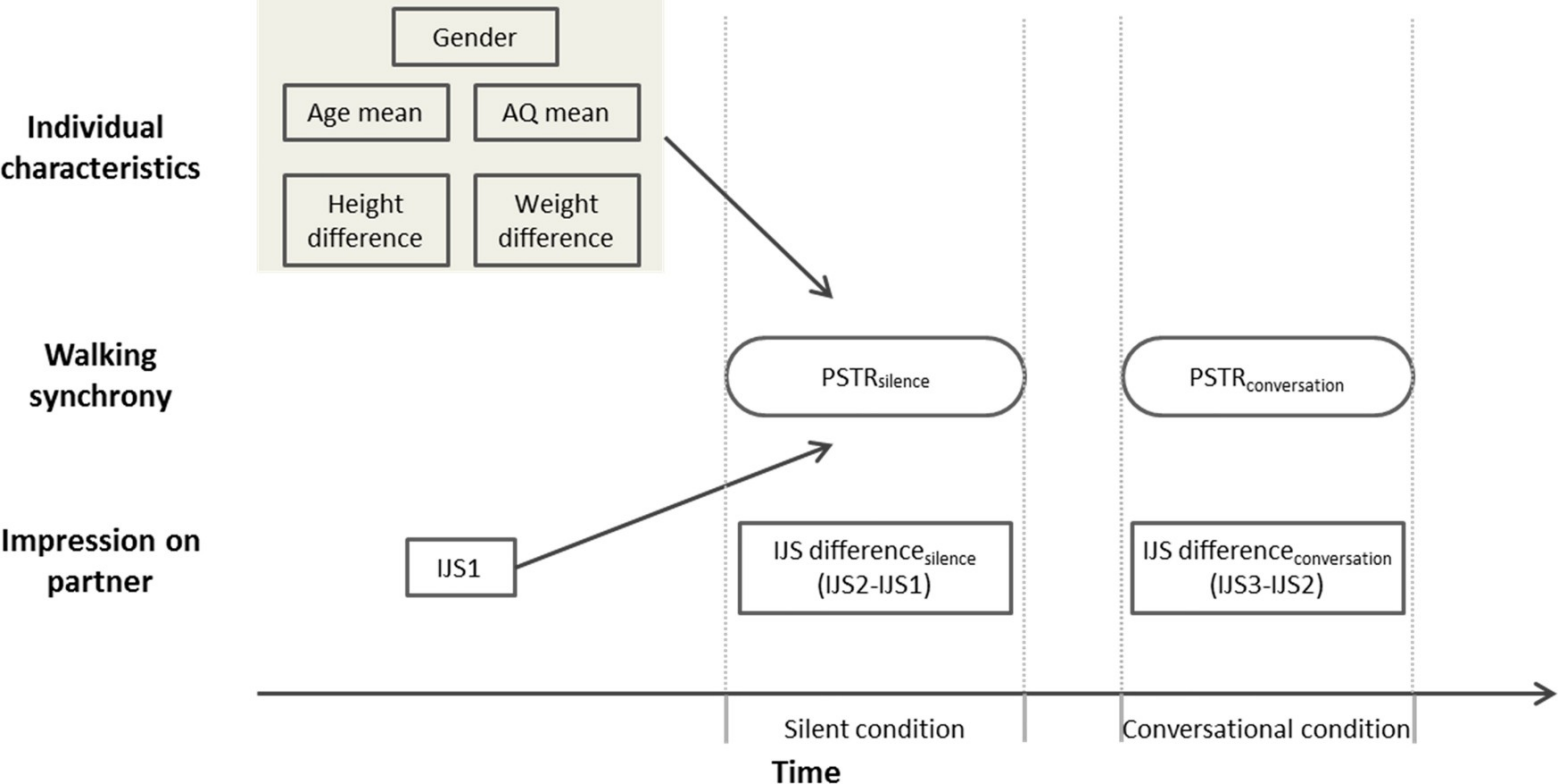
Possible factors that predicted the PSTR under the silent condition.

To enable us to control other known factors influencing synchrony, we re-examined whether the first impression effect remained once other factors had been considered. We conducted a generalized linear model (Fig 9) predicting the PSTR of the silent condition (PSTR_silence_) from the social relationship between walkers (IJS1) and individual characteristics (gender, mean age, mean AQ, height difference and weight difference of each pair). Three significant predicting effects were observed. IJS1 contributed significantly to predicting PSTR_silence_ (β = 0.035, p < .001), suggesting that walkers with a good first impression of each other tended to synchronize their steps more. The other two significant factors were gender (β = -0.290, p < .001) and height (β = 0.012, p = .002), implying that females and taller participants sync better in silent walk. Another generalized linear model analysis was applied to the PSTR of the conversational condition (see supporting data for a detailed report). Results did not show a significant predicting effect on PSTR_conversational_ from IJS1 (S1 Fig).

## Experiment 2: Control experiment without joint walking task

### Method

To test whether the IJS increment under the silent condition came from walking per se or simply spending time together, we performed a control experiment to investigate whether participants in a silent no-walking situation would increase impression rating for each other.

#### Participants

We estimated the sample size used in Experiment 1 in which we reported a significant IJS difference before and after a silent walk from a paired t-test with an effect size (Cohen’s d) of 0.366. Because the current study was conducted without conversation, we expected a similar effect size. Hence, with an estimated effect size of 0.366, a power analysis conducted using G*Power [24] showed that a sample size of 61 pairs was needed for a paired t-test to reach a power of 0.8 with the alpha level at 0.05. Ultimately, 64 pairs participated in this silence control experiment. All participants provided written informed consent before the experiment began. All the procedures were approved by the Tohoku University Human Research Ethics Committee and performed in accordance with the principles expressed in the Declaration of Helsinki as regards the treatment of participants.

#### Procedure

Participants were recruited and informed that they were to join a psychology experiment with an assigned partner. In reality, they only took part in the experiment alone. Seventeen participants (8 females, mean age 21.5 years old, SD 3.1 years old) were separated into two groups (8 and 9 members) and scheduled at two different time slots. At the beginning, participants were instructed to believe that later in the experiment, they would be paired with every other member in their group to perform some joint decision-making tasks. The seats were arranged in a circle so that they could see all others group members labeled with a single digit number (1 to 9). This seating arrangement was made to be similar to that in Experiment 1. Participants provide their first impression to each of their group members by completing the Interpersonal Judgement Scale (IJS) questionnaire after signing the consent form. This was similar to the scenario when participants filled their first impression at IJS-1, and they filled in the rating for each of them without a chance to talk or to know each other. At that time, they had no knowledge about the experiment design. They were then given questionnaires about their campus life and university activities to fill in. They were instructed to focus on their own task without paying attention to or conversing with the other person. It took about 5–7 minutes, similar to the walking duration in Experiment 1. After they return the questionnaires, they were invited to rate each other again using the IJS (IJS 2). Before they left, they were debriefed about the real purpose of the experiment.

In the group of 8 members, they formed totally 28 pairs. In the group of 9 members, they formed 36 pairs in total. Together, there were 64 pairs in Experiment 2.

### Results

**Fig 10:**
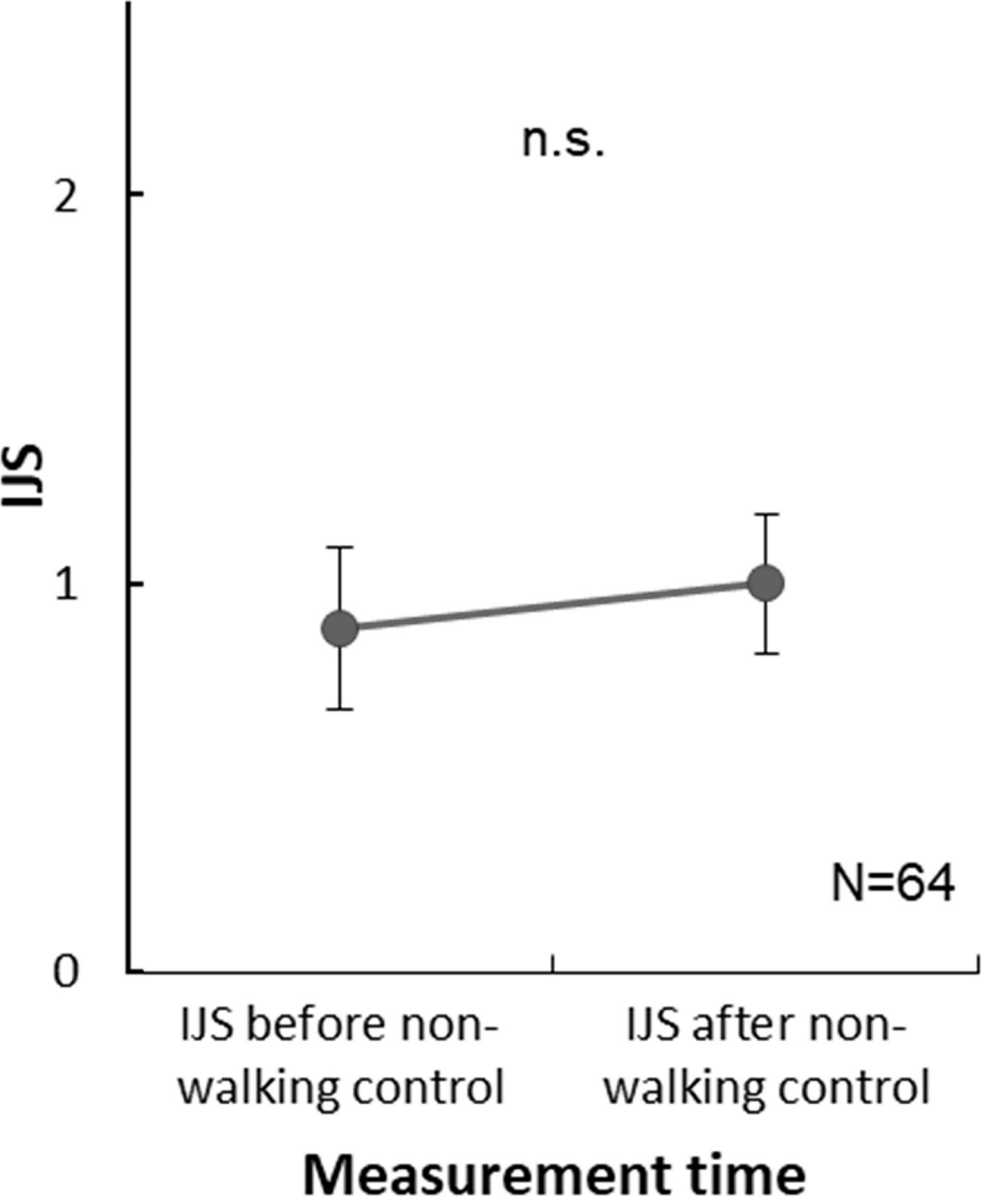
Means and 95% confidence intervals for paired participants’ total IJS scores measured before and after a non-walking control experiment (n.s., not significant).

The result of a paired t-test revealed no significant difference (p = .216) between IJSs before (0.883) and after (1.000) the task (Fig 10), indicating that the silent co-occupation of a space was insufficient to improve the interpersonal impression.

## Experiment 3: Control experiment with silent joint walking

### Method

In Experiment 1, IJS rating increased more in conversational condition than in silent condition. Was the additional increment caused by conversation or extra period of paired walking? To delineate these two possibilities, we conducted another control experiment identical to the main experiment except the talk with conversation was replaced with another silent walk.

#### Participants

We estimated the sample size used in Experiment 1 in which we reported a significant IJS difference before and after a silent walk from a paired t-test with an effect size (Cohen’s d) of 0.366. Because the current study was conducted without conversation, we expected a similar effect size. Hence, with an estimated effect size of 0.366, a power analysis conducted using G*Power [24] showed that a sample size of 61 pairs was needed for a paired t-test to reach a power of 0.8 with the alpha level at 0.05. Ultimately, 66 pairs participated in this silence control experiment. All participants provided written informed consent before the experiment began. All the procedures were approved by the Tohoku University Human Research Ethics Committee. All the methods were performed in accordance with the principles expressed in the Declaration of Helsinki as regards the treatment of participants.

#### Procedure

Participants were recruited and informed that they were to join a psychology experiment on time perception with an assigned partner. Each time, four participants were recruited to form 6 possible pairs. Forty-four participants (22 females, mean age 21.4 years, SD 2.9 years) joined to form 66 pairs in total. The procedure was identical to Experiment 1 except the pair never had any conversations. Participants provide their first impression to each of their group members by completing the Interpersonal Judgement Scale (IJS) questionnaire after signing the consent form (IJS-1) without a chance to talk or to know each other. Then they paired with each of the group member and walked with the assigned partner one by one (silent walk 1). At the turning point of the path, they stopped and reported their perceived duration of the walk. Then they rated each other again using the IJS questionnaire (IJS 2). After that, they walked back to the starting point (silent walk 2), where they reported the perceived duration and rated each other again using IJS (IJS3). After the experiment, they were debriefed about the real purpose of the experiment.

### Results

Two pairs’ results were excluded because they were acquaintances before the experiment. In total, 64 pairs were included in data analysis.

**Fig 11:**
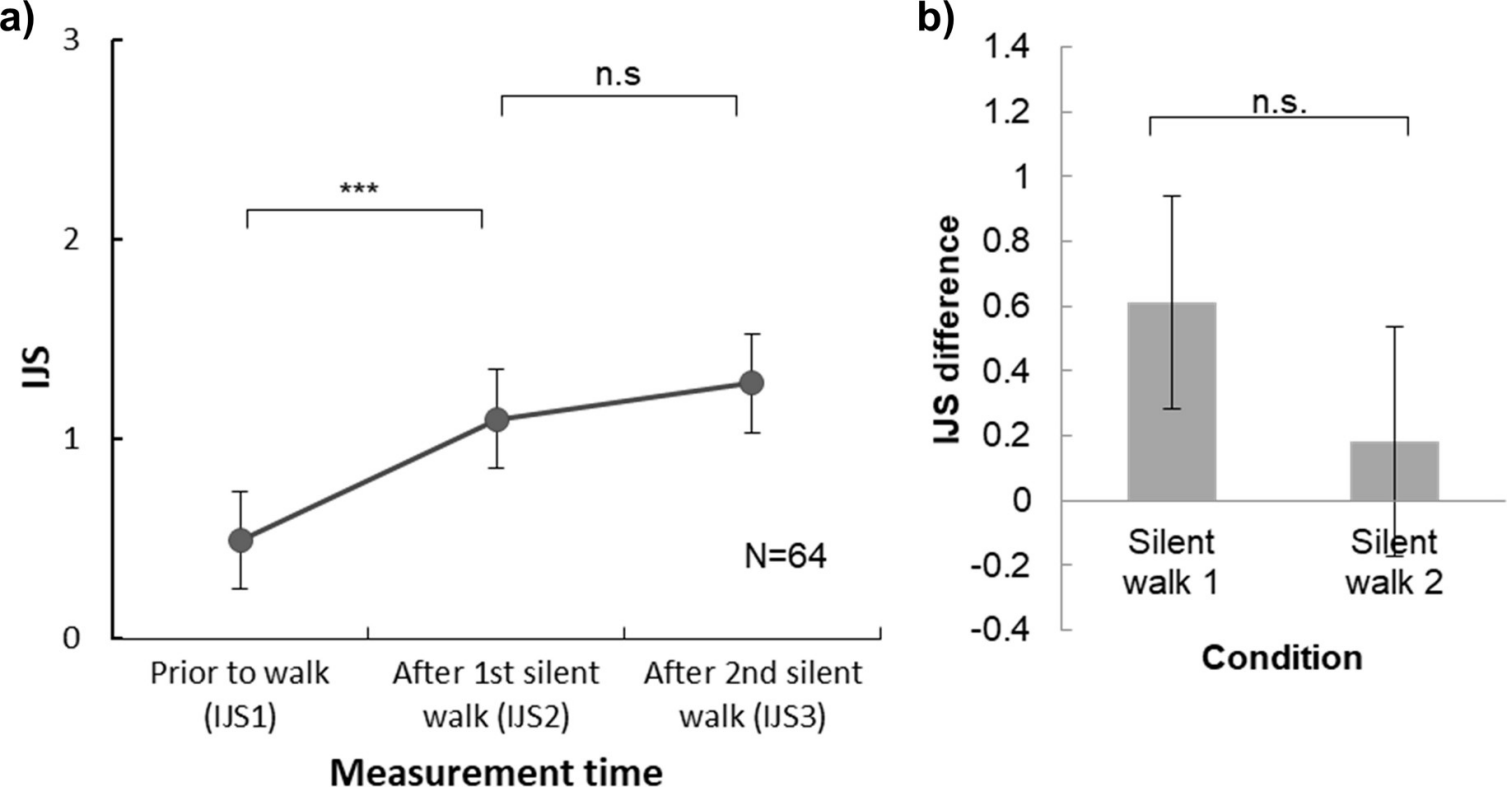
Results of impression rating. a) Means and 95% confidence intervals for total IJS scores measured before the silent condition (IJS1), after the 1^st^ silent walk (IJS2) and after the 2^nd^ silent walk (IJS3). b) IJS difference in 1^st^ silent (IJS2-IJS1) and 2^nd^ silent walks (IJS3-IJS2) (***, p < .001; n.s., not significant).

Results of a one-way repeated measure ANOVA test showed that IJS scores increased in both silent walks (Fig 11A). The main effect showed a significant difference among 3 IJS scores, F(2, 126) = 11.484, p < .001, η^2^ = .154. Post-hoc tests with Bonferroni adjustment showed that IJS1 (0.492) significantly increased after first silent walk (IJS2 = 1.102, p < .001), and maintained at a constant level after the second silent walk (IJS3 = 1.281, p = .974).

Then we obtained the IJS difference of the first silent walk (by subtracting IJS1 scores IJS2 scores) and second silent walk (by subtracting IJS2 scores from IJS3 scores) (Fig 11B). A paired t-test was applied and showed that IJS increment after the first silent walk (0.609) did not differ from that from the second silent walk (0.180, t(63) = 1.998, p = 0.164).

In summary, participants increased impression rating less in the second silent walk than in the first silent walk. In Experiment 1 where the second walk allowed conversation, the impression increment was higher than that in the first silent walk, suggesting the extra impression increment was benefited from conversation.

## Discussion

In this study, we disassociated the contribution of verbal communication from walking step synchronization and discovered that with no conversation taking place, paired strangers unconsciously synchronized theirs steps. Their mutual impression was more favorable after the silent walk. Our analysis revealed a unidirectional relationship between synchrony and impression rating: a better first impression led to a higher walking synchrony between two strangers walking side by side in silence, but their step synchronization did not modulate the later impression increment. Moreover, the first impression effect on synchrony was observed only in female participants.

The first impression plays a role in promoting implicit walking synchrony. Many studies have reported that interpersonal motor synchronization increases liking[6–9]. However, less is known about the opposite direction, i.e. whether liking enhances motor synchrony. Previous research has indirectly indicated that the likability of a partner is an antecedent for motor synchrony. For instance, people are more likely to synchronize with an attractive partner [1] and a punctual partner [3]. Zhao et al. [2] directly investigated the effect of likability on body synchrony and reported that participants showed more synchrony in a finger tapping task with a more likable partner. Consistently, the current study supports the view that a positive first impression boosts body synchronization in another form of joint action, namely paired walking. The commonality of the above antecedents of synchrony (attractiveness, punctuality, and likability) is that they create a positive impression of the partner. And a good impression is known to promote the establishment of rapport in interpersonal activities, such as job interviews [31, 32] and sales encounters [33]. It is possible that a good impression elicits a willingness to interact explicitly and implicitly, which adjusts a dyad’s coordination in a synchronous fashion.

The social impression increment in silent walk condition does not correlate with walking synchrony. This appears to be inconsistent with a recent finding, namely that participants showed a larger impression increment for a synchronized partner during paired walking with conversation [18]. It is possible that the impression increment during silent walking was too subtle to observe. The difference between the IJSs pre- and post-walk under the conversational condition in Cheng et al. [18] was almost five times that under the silent condition (2.137 vs. 0.447). The small impression increment range might have made the interaction between impression increment and walking synchrony difficult to detect. Another possible explanation is that to shape a social impression, implicit body synchronization needs the help of explicit cues such as explicit verbal communication and perceived body coordination. This speculation is inspired by recent reports of interactions between other implicit-explicit dissociated processes. For example, Park and Donaldson [34] reported that the implicit priming effect was larger on items that were successfully recognized (i.e. explicit memory), suggesting a novel functional role in memory collection. Seitz and Watanabe [35] found that when participants were exposed to a display consisting of a group of randomly moving dots that contained a small group of dots moving in the same direction but invisibly, their detection thresholds toward this direction gradually improved without their knowledge (implicit learning). However, this learning only occurred when the participants’ attention was engaged in another irrelevant central task, which suggested that top-down influences penetrated implicit processes. Similarly, when we review past findings on synchrony-produced liking, many observations were made when the participants were engaged in conversation [17, 18] or when they were repeatedly performing noticeable synchronous actions [6–9]. Explicit interaction may be a necessary medium for realizing implicit synchrony and thus shape social relationships. One working hypothesis is that when people interact explicitly through a noticeable exchange of information (e.g. conversation or perceived motor coordination), implicit synchrony imposes additional modulation on a social relationship; when there is no explicit interaction (e.g. walking silently), modulation from implicit synchrony becomes inactive. This hypothesis awaits further testing via empirical studies.

**Fig 12:**
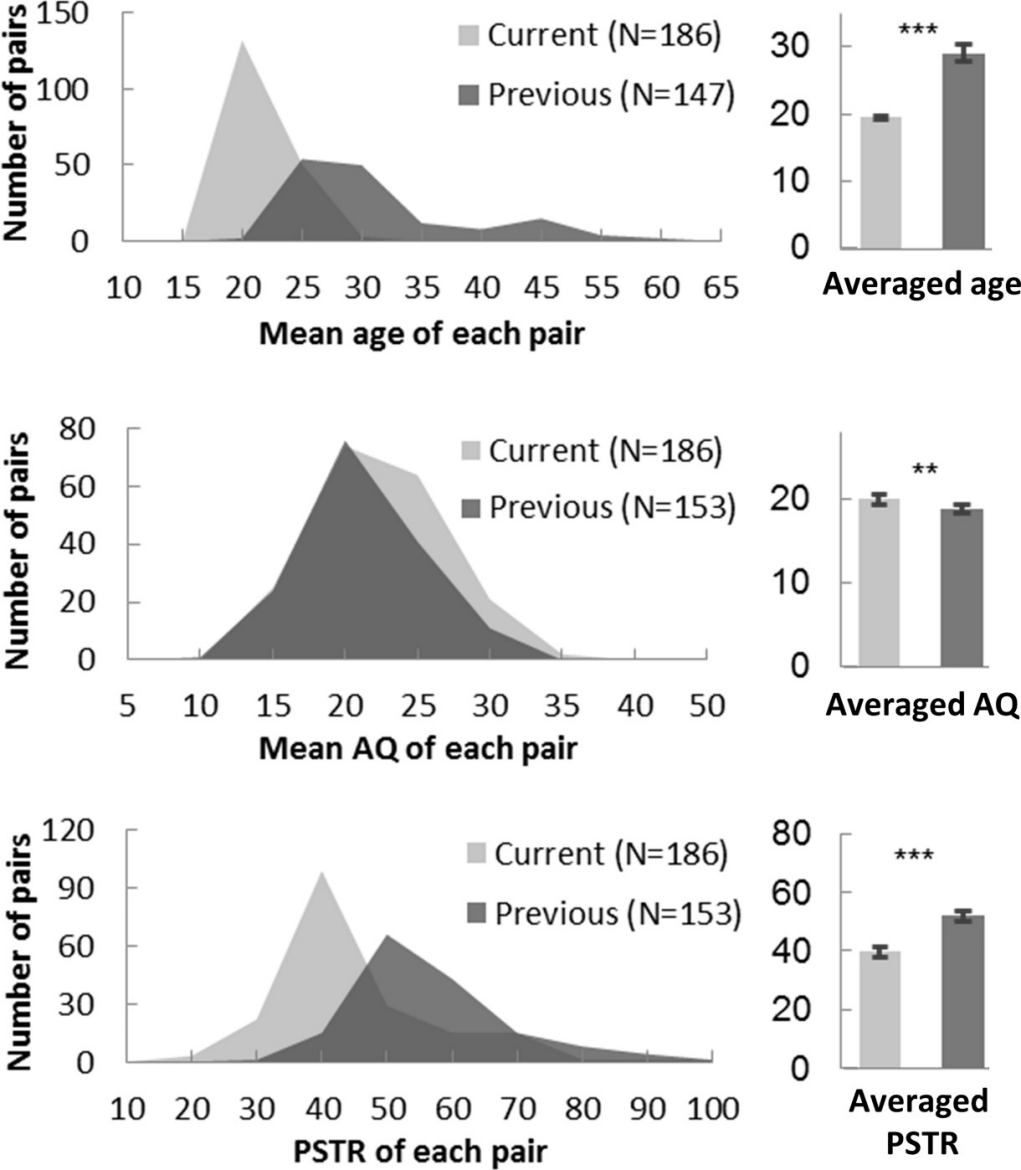
Distribution of mean age, mean AQ and PSTR for participants in the current and previous studies.

Regarding individual differences (i.e. gender and autistic traits), we observed that only gender predisposed walking synchrony in the current study. The absent AQ effect was inconsistent with our previous results [18] at first look. On closer inspection, we found that the participants (N = 153 stranger pairs) in the current study were significantly younger (19.758 years old, t(331) = 16.135, p < .001), more autistic (20.888, t(337) = 2.686, p = .008), and walked in a less synchronized manner (PSTR, 39.648; t(337) = 9.380, p < .001) than those (N = 186 pairs) in Cheng et al. [18] (average age, 29.245; average AQ, 18.882; PSTR, 51.979) (Fig 12). To take advantage of a bigger sample and cover a greater variety of participants, we combined the samples from these two studies and employed a Generalized Linear Model to predict the PSTR using individual characteristics (gender, mean age, mean AQ, height difference and weight difference of each pair) and impression ratings (IJS before walk). Gender (β = -0.352, p < .001), age (β = 0.014, p < .001) and AQ (β = -0.007 , p < .001) were discovered to contribute significantly when predicting the PSTR, suggesting that females showed higher synchrony than males and older and less autistic walkers tended to synchronize more. With a bigger and a more representative sample size, we replicated the AQ effect and found a new effect of age on synchrony. The narrow age and AQ ranges of the participant sample in the current study might have limited our ability to detect their effects on participants’ walking synchrony.

The mechanism underlying the age effect in implicit interpersonal coordination is intriguing but remains unclear. Higher motor synchrony is related to better motor coordination skills [9], prosocial attitudes [4], agreement and more speech convergence in conversation [10, 21], and a higher perceived similarity between partners [5]. It is possible that older participants have advantages with respect to one or more of the qualities mentioned above. For example, in terms of motor coordination skills, developmental studies of early human life show that babies’ movements in coordination with their mothers started to appear from 3 months old and the tendency increases during infancy [36, 37]. Children are able to coordinate with partners in tempo tasks for the age of 2 [38]. Interpersonal coordination skills in various tasks improve across childhood (2–4 years old: [38], 6–9 years old: [39], 6–12 years old: [40]). Individual motor skills also improve throughout childhood and adolescence and are associated with physical activity ([41, 42], also see a review article, [43]). For those above 60 years of age, obvious motor deficits were observed [44, 45]. However, to the best of our knowledge, there has been no systematic research on developmental changes in motor coordination across the entire lifespan (e.g. our tested group had an age range of between 18 and 60 years old). This made it hard for us to speculate on an age effect from motor coordination skills. Regarding prosocial attitudes, there is evidence showing that the prosocial personality generally increases from late adolescence to the early 20s (17–18 to 21–22 years old) [46] and remains stable in early adulthood (21–26 years old) [47]. As the prosocial personality stabilizes in early adulthood, it is unlikely that this is the main factor accounting for the effect of age on walking synchrony. With respect to explicit communication, older adults have poorer conversation skills and communication efficiency due to their reduced ability to decode linguistic information and maintain content details in the population aged from 18 to 90 years old (see a review article, [48]). This contradicts our expectation that older adults tend to create a better social context in which to increase walking synchrony. In terms of implicit coordination, there is no known study examining whether or not older people excel in implicit synchrony or other forms of interpersonal coordination (e.g. automatic nonverbal mimicry, emotional contagion, and speech convergence). Our study provides new insights into the lifespan development of implicit motor synchrony and future investigations exploring the age effect should be encouraged.

There is a growing awareness of the validity of interpersonal interaction research in real world scenarios [49, 50]. Motor synchronization has been widely studied in a laboratory environment (see a review article: [51]). To make it possible to apply laboratory findings to robotic design and other applications, it is important to investigate how people interact in daily life. However, daily natural environments are rich in their contextual information, which makes experiment control a challenge. Therefore, it is important to consider possible confounding variables and retest the paradigm to ensure scientific reliability. We previously studied walking synchrony in the most common daily situation, that is paired walking while chatting [18]. In the current study, we further isolated implicit body synchrony from explicit communication by removing conversation. Our results revealed that implicit synchrony was shaped by the first impression, which was not observed in previous walk-and-chat tasks. The new finding benefited from our efforts to balance the trade-off between ecological validity and scientific control.

Walking synchrony may be one form of interpersonal communication in a larger category of implicit social interaction. There is another similar type of nonverbal communication that is closely associated with social factors: automatic mimicry (i.e. spontaneous/automatic imitation). It refers to the spontaneous imitation of body movements (e.g. face rubbing and foot shaking) and facial expressions [52]. Both body synchrony and automatic mimicry involve the matching of interpersonal nonverbal behavior. The difference is that the former emphasizes the occurrence of temporal motion and the latter focuses less on temporal dynamics and more on the repetition of the same behavior. A similar phenomenon to body synchrony, namely automatic mimicry, also plays an important role in social interaction [53, 54]. On one hand, social factors shape spontaneous mimicry. For example, people tend to mimic a partner who is attractive [55] or an ingroup member [56]. On the other hand, automatic mimicry boosts positive social consequences [53, 54], including liking [52], rapport [57], and prosocial behavior [58, 59]. Research on body synchrony and automatic mimicry reveal that implicit communication and social factors are interwoven, which suggests an essential role for implicit communication in social interaction. The crucial role of unconscious processes is widely recognized in the cognitive sciences, and these affect how people perceive, memorize and process information, and form decisions [60]. Nevertheless, we have little understanding about how unconscious processes smooth social interaction. Future efforts should strive to understand how implicit interpersonal information provides a basis for effective communication via multi-channels, such as sensorimotor coordination and synchrony in physiological and neural processes [61].

In summary, this study has methodological and theoretical implications for research on implicit communication. We provide an approach with which to study implicit walking synchrony while disassociating it from verbal communication. Furthermore, we discover that a liking between partners, which is known to be a consequence of synchrony, also predisposes synchrony. Together with previous findings on social consequences from synchronous motion, this bidirectional relation indicates the crucial role of implicit communication in social interaction.

## Supporting information

S1 Fig Possible factors that predicted the PSTR in the conversational condition. We conducted a generalized linear model as regards predicting the PSTR of the conversational condition (PSTR_conversation_) based on the social relationship between walkers (IJS1, the difference between IJS2 and IJS1) and individual characteristics (gender, mean age, the mean AQ, height difference and weight difference of each pair). The first impression (IJS1) did not significantly contributed to predicting PSTR_conversation_ (β = 0.022, p = .056). Among all factors, four showed significant effect. Females synchronized steps better than males (β = -0.344, p < .001). Age significantly predicted PSTR_conversation_ (β = -0.036, p < .001), showing that younger pairs synchronized better. PSTR_conversation_ positively correlated with height (β = 0.018, p < .001) and weight (β = 0.006, p = .033).
(TIF)
