# Supplementary figures and images for: Paired walkers with better first impression synchronize better
Source: PLoS One. 2020 Feb 21;15(2):e0227880. doi: 10.1371/journal.pone.0227880 (PMC7034894; doi:10.1371/journal.pone.0227880)

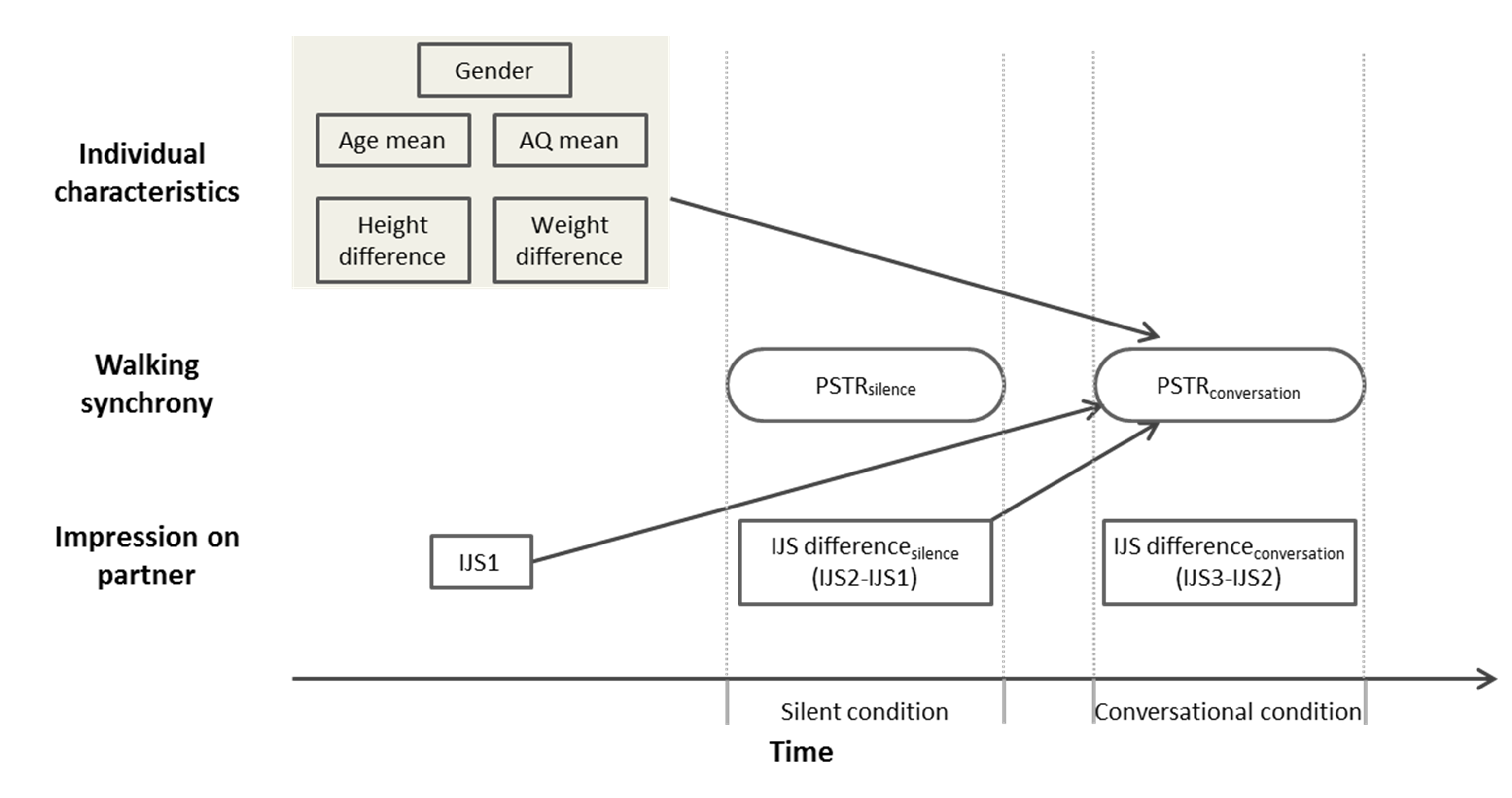

Supplement: S1 Fig — We conducted a generalized linear model as regards predicting the PSTR of the conversational condition (PSTRconversation) based on the social relationship between walkers (IJS1, the difference between IJS2 and IJS1) and individual characteristics (gender, mean age, the mean AQ, height difference and weight difference of each pair). The first impression (IJS1) did not significantly contributed to predicting PSTRconversation (β = 0.022, p = .056). Among all factors, four showed significant effect. Females synchronized steps better than males (β = -0.344, p < .001). Age significantly predicted PSTRconversation (β = -0.036, p < .001), showing that younger pairs synchronized better. PSTRconversation positively correlated with height (β = 0.018, p < .001) and weight (β = 0.006, p = .033). (TIF) [file pone.0227880.s001.tif]
